# Targeting the anaphase-promoting complex/cyclosome (APC/C)- bromodomain containing 7 (BRD7) pathway for human osteosarcoma

**DOI:** 10.18632/oncotarget.1816

**Published:** 2014-03-21

**Authors:** Kaishun Hu, Dan Liao, Wenjing Wu, An-Jia Han, Hui-Juan Shi, Fen Wang, Xin Wang, Li Zhong, Tingmei Duan, Yuanzhong Wu, Jingying Cao, Jianjun Tang, Yi Sang, Li Wang, Xiaobin Lv, Shuangbing Xu, Ru-Hua Zhang, Wu-Guo Deng, Sheng-Ping Li, Yi-Xin Zeng, Tiebang Kang

**Affiliations:** ^1^ State Key Laboratory of Oncology in South China, Collaborative Innovation Center for Cancer Medicine, Sun Yat-Sen University Cancer Center, Guangzhou, China; ^2^ Department of Pathology, First Affiliated Hospital, Sun Yat-Sen University, Guangzhou, China

**Keywords:** APC/C, BRD7, Cancer Target

## Abstract

Osteosarcoma is the most common primary malignant bone tumor in childhood and adolescence and has a propensity for local invasion and early lung metastasis. However, the current therapies often result in chemoresistance, and a therapeutic target is not available in the clinic for osteosarcoma. Here, we report that BRD7 forms a complex with the anaphase-promoting complex/cyclosome (APC/C) and is degraded by APC/C^cdh1^ and APC/C^cdc20^ during the cell cycle. Moreover, BRD7 is a tumor suppressor in osteosarcoma, and the BRD7 mutant resistant to degradation by APC/C is more efficient than the wild-type protein at suppressing proliferation, colony formation, and tumor growth of osteosarcoma *in vitro* and *in vivo*. The combination of proTAME, an inhibitor of APC/C, with chemotherapeutic drugs efficiently targets osteosarcoma *in vitro*. Furthermore, there is a strong inverse correlation of protein levels between BRD7 and Cdh1 or Cdc20, and lower BRD7 expression is an indicator for poor prognosis in patients with osteosarcoma. Collectively, our results indicate that targeting the APC/C-BRD7 pathway may be a novel strategy for treating osteosarcoma.

## INTRODUCTION

The progression of the cell cycle, particularly mitosis, is mainly regulated by the anaphase-promoting complex/cyclosome (APC/C), a multisubunit member of the RING finger family of ubiquitin ligases. APC/C is composed of many different subunits, including APC1-8, APC9-11, and CDC26 [1, 2], and recognizes its substrates through two adaptor proteins, Cdh1 and Cdc20, which also serve as co-activators for APC/C at different phases of mitosis. In early mitosis, APC/C binding to Cdc20 leads to the initiation of anaphase. By contrast, association with Cdh1 in late mitosis maintains APC/C activity throughout the subsequent G1 phase [3-6]. APC/C mediates ubiquitination and degradation of many critical proteins that have distinct functions during mitotic exit, including Aurora-A and -B, cyclins-A and -B, survivin, Plk1, Nek2A, and securin [7]. However, novel substrates of APC/C during mitotic exit have also been frequently identified, facilitating our understanding of the roles and mechanisms of APC/C and/or mitosis.

Bromodomain containing 7 (BRD7), also known as BP75, NAG4, or CELTIX1, is ubiquitously expressed in human tissues [8]. Recent studies have revealed that BRD7 is mainly located in the nucleus and modulates chromatin remodeling by binding to acetylated histone H3 [9, 10]. Accumulating evidence indicates that BRD7 may serve as a tumor suppressor. For example, BRD7 was down-regulated in nasopharyngeal carcinoma, prostate cancer and epithelial ovarian carcinoma and could inhibit cell growth through multiple mechanisms, including cell cycle arrest [11-13]. Interestingly, BRD7 interacts directly with p53 and is essential for the transcriptional activation of p53 target genes such as p21, HDM2(MDM2) and TIGAR [13,14]. Collectively, BRD7 may play important roles in the progression of the cell cycle, however, during which BRD7 regulation has not yet been explored.

Osteosarcoma is a rare type of tumor with poor prognosis in childhood and adolescence[15], with a propensity for local invasion and early lung metastasis. Currently, the five-year survival rate remains at approximately 65% to 70% for localized disease but drops to only 20% for metastatic disease, with modest therapeutic improvement over the past 15 years [16,17]. These outcomes are mainly due to the limited effectiveness of current therapies that often result in chemoresistance. Therefore, it is urgent to identify new therapeutic targets for osteosarcoma [18]. In this report, we demonstrate that BRD7, as a new substrate of APC/C-E3 ligase during the cell cycle, is a tumor suppressor in osteosarcoma, and this APC/C-BRD7 pathway may provide a potential therapeutic target for treating osteosarcoma.

## RESULTS

### BRD7 may be a new substrate of APC/C E3 ligase during mitotic exit in osteosarcoma cells

**Fig 1:**
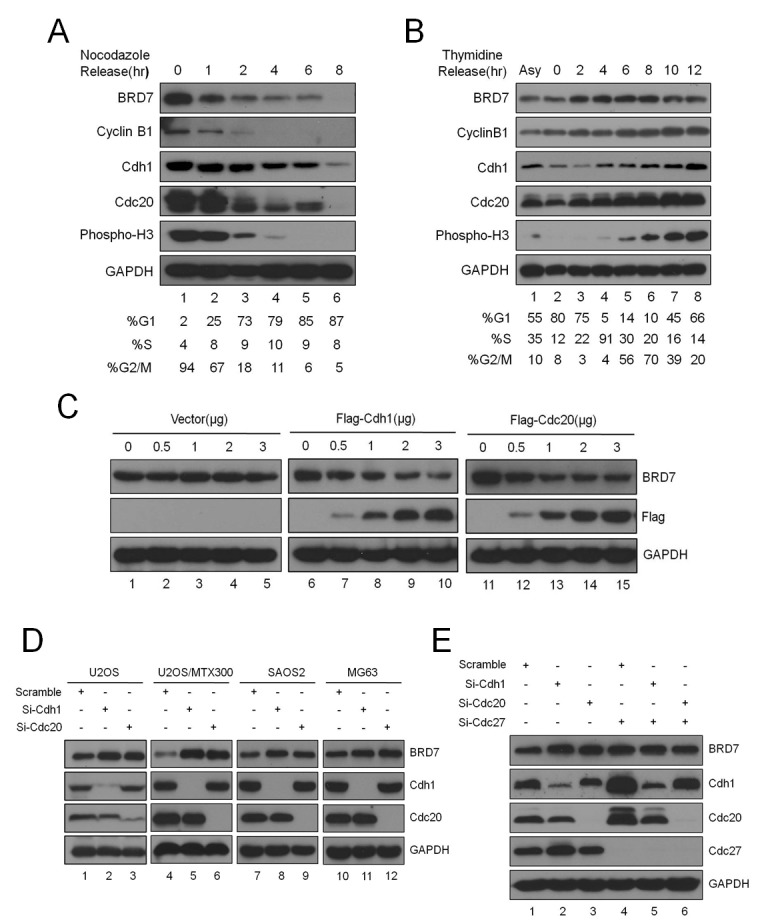
BRD7 is a substrate of APC/C E3 ligase during mitotic exit in osteosarcoma cells (A). U2OS cells were arrested in mitosis following 18 hrs of nocodazole treatment, released into fresh medium, and collected at the indicated times. The cells were divided into three parts: one for Western blot analysis with the indicated antibodies, one for analysis by flow cytometry, and one for mRNA measurement (Fig. S1) (n=3). (B). U2OS cells were blocked at the G1/S boundary by double thymidine treatment, released into fresh medium, and harvested at the indicated times. The cells were divided into two parts: one for Western blot analysis with the indicated antibodies and the other for flow cytometry analysis(n=3). (C) U2OS cells were transiently transfected with the indicated concentrations of empty vector, Cdh1 or Cdc20 expression plasmids for 30 hrs and evaluated for GAPDH, BRD7, Cdh1, and Cdc20 protein levels by Western blotting (n=3). (D) The four osteosarcoma cell lines indicated were transiently transfected with scrambled siRNA, Cdh1 siRNA, or Cdc20 siRNA for 48 hrs and were analyzed as in(C) (n=3). (E) U2OS cells were transfected with siRNA, Cdh1 siRNA or Cdc20 siRNA alone, or together with Cdc27 siRNA. After incubation for 48 hrs, the cells were analyzed as in (C) (n=3).

Previous studies have shown that BRD7 may play important roles in the progression of the cell cycle [11, 12, 19]. However, studies concerning the regulation of BRD7 during the cell cycle are lacking. Considering that the BRD7 protein sequence contains one D-box (RxxL) and one KEN-box, which are the typical motifs recognized by APC/C E3 ligase [20, 21], a key regulator for mitotic exit [1, 2], we sought to determine whether BRD7 is a substrate of APC/C. To this end, U2OS cells were arrested at metaphase by nocodazole and then released. As shown in Fig. 1A, the BRD7 protein gradually decreased during mitotic exit, as indicated by mitotic proteins such as cyclinB1, Cdc20, Cdh1 and phospho-Histone 3, whereas the mRNA level of BRD7 remained constant during the release (Fig. S1). Consistently, as shown in Fig. 1B, the BRD7 protein accumulated gradually during G1 and S, peaked at G2/M, and decreased again after mitosis using U2OS cells that were synchronized at the G1/S boundary and then released. These results demonstrated that the BRD7 protein level fluctuated during the cell cycle, and BRD7 is degraded during mitotic exit, indicating BRD7 may be a potential substrate of APC/C.

Indeed, overexpression of Cdh1 or Cdc20, the APC/C activators, reduced endogenous BRD7 in a dose-dependent manner (Fig. 1C), whereas knockdown of endogenous Cdh1 or Cdc20 using siRNA resulted in the up-regulation of endogenous BRD7 in U2OS cells (Fig. 1D). Furthermore, as shown in Figs. 1E and S2, the knockdown of endogenous Cdc27 or APC2, both of which are core subunits of the APC/C E3 ligase complex, by siRNA also increased BRD7 protein, and this increase was not further enhanced by the knockdown of endogenous Cdh1 or Cdc20. Interestingly, this up-regulation of BRD7 by the knockdown of Cdh1 or Cdc20 appears to be specific in osteosarcoma cell lines, including U2OS and its derivative line U2OS/MTX300, as well as SAOS2 and MG63 cells (Fig. 1D), because this phenomenon was not observed in other cell lines such as HeLa, HepG2, HCT116, MCF7 and CNE2 (Fig. S3). Consistently, HeLa cells were arrested at metaphase by nocodazole and then released, the protein level of BRD7 remained constant during mitotic exit, as indicated by mitotic proteins such as cyclinB1, Cdc20 and Cdh1 (Fig. S4). Taken together, the data show that BRD7 may be degraded by APC/C E3 ligase during mitotic exit in osteosarcoma cells.

### Both the D-box and the KEN-box are required for the degradation of BRD7 by APC/C E3 ligase

**Fig 2:**
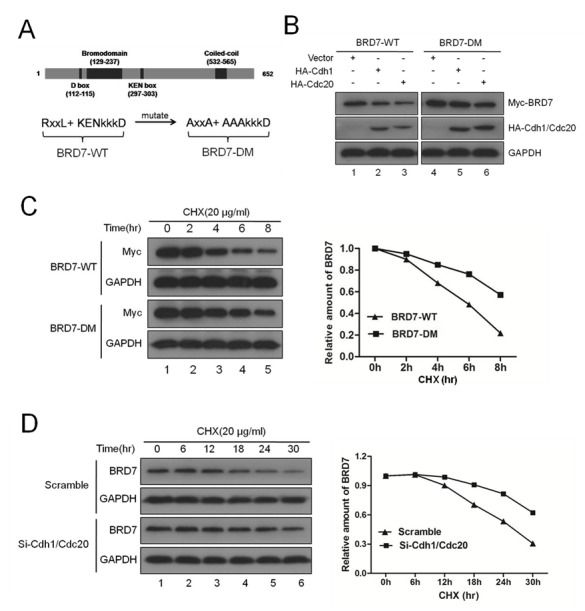
Both the D-box and the KEN-box are required for the degradation of BRD7 by APC/C E3 ligase (A) Schematic illustration of BRD7-WT and its double mutant BRD7-DM. (B) U2OS cells were transfected with Myc-BRD7 wild-type (BRD7-WT) or its double mutant (BRD7-DM) with empty vector, HA-Cdh1 or HA-Cdc20 for 30 hrs, and then were subjected to Western blotting with the indicated antibodies (n=3). (C) U2OS cells were transfected with Myc-BRD7 wild-type (BRD7-WT) or its double mutant (BRD7-DM) as indicated for 24 hrs, and then were treated with 20 µg/ml cycloheximide (CHX) for the indicated times. The cells were then analyzed by Western blotting (left panel), and the densities of the BRD7 protein bands at each time point were normalized to GAPDH and were calculated into percentages using 100% as the value of the zero time point (right panel). (D) U2OS cells were transfected with scrambled siRNA, or Cdh1 siRNA and Cdc20 siRNA for 48 hrs, and then were incubated with 20 µg/ml CHX for the indicated times. The cells were then analyzed as described in (C) n=3.

Next, we generated the BRD7 double mutant (DM), in which both the D-box (RxxL) and the KEN-box in BRD7 were mutated into alanine (A) residues as illustrated in Fig. 2A. As expected, BRD7-DM was marginally altered, whereas BRD7-WT was obviously decreased when Cdh1 or Cdc20 was co-transfected into U2OS cells (Fig. 2B). Furthermore, the half-life of BRD7-DM was much longer than that of BRD7-WT (Fig. 2C), and endogenous BRD7 became more stable after the knockdown of both Cdh1 and Cdc20 using siRNA (Fig. 2D). These results revealed that APC/C^Cdh1^ or APC/C^cdc20^ degrades BRD7 through recognition of both the D-box (RxxL) and the KEN-box.

### BRD7 interacts with Cdh1 or Cdc20

**Fig 3:**
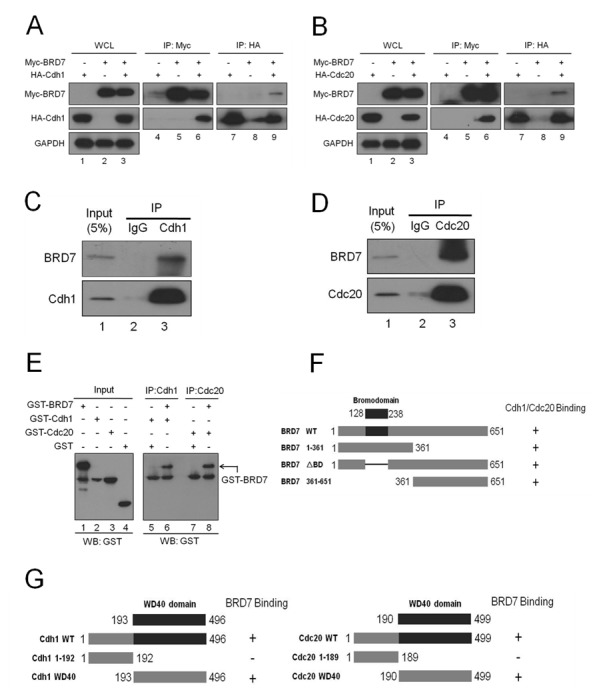
BRD7 interacts with Cdh1 or Cdc20 (A) and (B). U2OS cells transfected with the indicated plasmids for 24 hrs were lysed. Immunoprecipitation (IP) using anti-Myc or anti-HA agarose and Western blotting with the indicated antibody were performed. WCL, whole cell lysate. (C) and (D). U2OS cells were lysed and were subjected to IP using anti-immunoglobulin G, anti-Cdh1, or anti-Cdc20 as indicated, and then were analyzed by Western blotting. (E) GST, GST-Cdh1, GST-Cdc20 and GST-BRD7 expressed and purified from bacteria were directly analyzed by Western blotting (lanes 1-4); anti-Cdh1 or Cdc20 antibodies were used to immobilize GST-Cdh1 or GST-Cdc20 onto protein A/G beads and were incubated with GST or GST-BRD7 as indicated. The beads were then washed five times and analyzed by western blotting using anti-GST antibodies. (F) Schematic illustration of BRD7 WT and its mutants, as well as their binding abilities with Cdh1 or Cdc20, are indicated. (G) Schematic illustration of Cdh1 WT and its mutants, or Cdc20 WT and its mutants, as well as the binding ability for each with BRD7, are indicated.

Based on the functions of Cdh1 or Cdc20 in the APC/C E3 ligase complex, we sought to test whether BRD7 binds to Cdh1 or Cdc20. First, Myc-tagged BRD7 was co-transfected with HA-tagged Cdh1 or Cdc20 into U2OS cells, and reciprocal co-immunoprecipitation (IP) using anti-Myc or anti-HA was performed. As shown in Fig. 3A and 3B, the complex containing these two proteins was obviously detected in the cell lysates. In addition, as shown in Fig S5, the mutant BRD7 (BRD7-DM) resistant to degradation by APC/C had the same binding affinity with Cdh1 or Cdc20 compared to wild type BRD7 (BRD7-WT). Second, as shown in Fig. 3C and 3D, the endogenous complex between BRD7 and Cdh1 or Cdc20 was clearly detected using co-IP with anti-Cdh1 or anti-Cdc20 antibody in U2OS cells. Third, this interaction between BRD7 and Cdh1 or Cdc20 appears to be direct because recombinant GST-BRD7 binds to recombinant GST-cdh1 or GST-cdc20 *in vitro* (Fig. 3E). Collectively, these results indicate that BRD7 may physically interact with Cdh1 and Cdc20 *in vitro* and *in vivo*.

Next, a series of deletions of BRD7, Cdh1 or Cdc20 were generated (Fig. 3F and 3G) and co-IP was performed to map the binding domains of BRD7 with Cdh1 or Cdc20. As shown in Figs. 3F, S6A and S6B, the N-terminus (residues 1-361), the C-terminus (residues 361-651) and the mutant lacking the bromodomain (BRD7ΔBD) of BRD7 displayed similar binding affinities to Cdh1 or Cdc20, indicating that the bromodomain of BRD7 is not required for such an interaction of BRD7 with Cdh1 or Cdc20. However, as shown in Figs. 3G, S6C and S6D, the WD40 domains of Cdh1 or Cdc20 were necessary and sufficient to bind BRD7. These results further confirmed that BRD7 physically and specifically interacts with Cdh1 or Cdc20 in cells.

### The degradation of BRD7 by APC/C E3 ligase plays key roles in cell growth and the tumorigenesis of osteosarcoma

BRD7 has been documented to function as a tumor suppressor in several tumor types[10-13]. Thus, we tried to determine whether this is the case in osteosarcoma. To avoid off-target effects, three different siRNA duplexes specifically targeting different BRD7 coding regions were used, and knockdown of BRD7 significantly increased the percentage of U2OS cells at S-phase but decreased the percentage of these cells at G1-phase (Fig. S7A). Moreover, this alteration of the cell cycle profile by BRD7 siRNA in U2OS cells was completely rescued by co-transfection of the BRD7-WT or -DM expression plasmid, both of which are siRNA resistant, as shown in Fig. S7B. This finding is consistent with the report showing that BRD7 inhibited the G1/S transition in nasopharyngeal carcinoma[10,11], indicating that BRD7 may also be a tumor suppressor in osteosarcoma.

**Fig 4:**
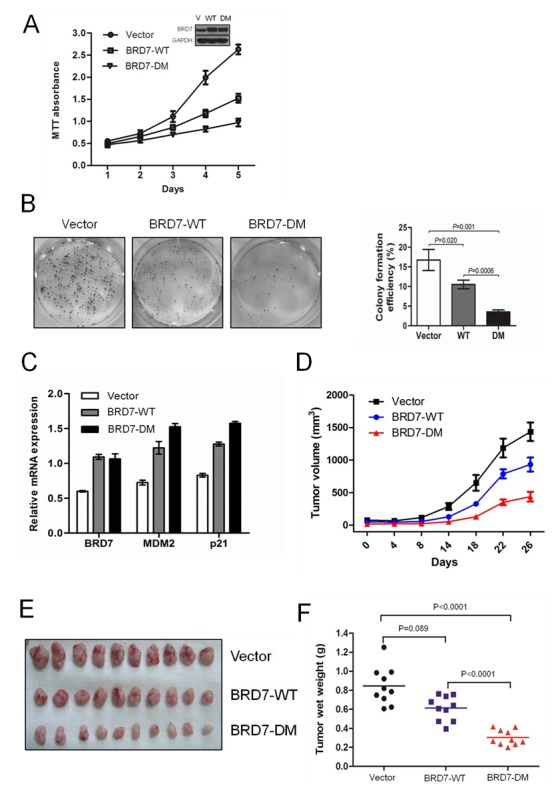
The degradation of BRD7 by APC/C E3 ligase plays key roles in cell growth and the tumorigenesis of osteosarcoma (A) U2OS cells were stably transfected with the indicated plasmids, as shown by Western blotting (insertion panel), and the cells were subjected to the MTT assay and graphed at the indicated times. The dots represent the mean, while the bars indicate the SD. * P <0.05 and ** P <0.001 using Student's t-test. (B) The stable transfectants used in (A) were cultured for 12 days, and the number of colonies was counted and graphed. P values were obtained by Student's t-test. (C) The stable cell lines used in (A) were subjected to qRT-PCR using primers specific for the indicated genes. (D)(E)(F) U2OS/MTX300 cells stably expressing empty vector, BRD7-WT, or BRD7-DM were injected into nude mice (2 × 10^6^ cells per mouse), and the tumor volumes were measured and recorded at the indicated time points. Additionally, tumor growth curves were created for each group (n = 10). The dots represent the mean, while the bars indicate the SD. * P<0.05 and ** P<0.001 using Student's t-test. After 6 weeks, the xenografts were excised from the mice and weighed.

Next, U2OS/MTX300 cells stably expressing empty vector, BRD7-WT, or BRD7-DM were generated, and MTT and colony formation assays were performed. As shown in Fig. 4A and 4B, the growth of transfectants with BRD7-WT was much slower than that of transfectants with vectors, supporting that BRD7 functions as a tumor suppressor in osteosarcoma. More strikingly, the transfectants with BRD7-DM grew much slower than those with BRD7-WT, demonstrating that BRD7-DM has a stronger capability of inhibiting cell growth than BRD7-WT. The cause may be that BRD7-DM is resistant to degradation by APC/C E3 ligase and may become more stable than BRD7-WT. Indeed, as shown in Fig. 4C, BRD7-DM had a higher activity to increase the mRNA levels of p21 and MDM2, two targets of p53, because BRD7 is required for the transcriptional activation of a subset of p53 target genes, such as p21 and MDM2 [13]. Furthermore, using the xenograft nude mouse model with the same stable U2OS/MTX300 transfectants, mice injected with BRD7-DM had the smallest tumor weights, whereas smaller tumor weights were detected in mice injected with BRD7-WT than those injected with vector (Fig. 4D, 4E, 4F and Fig. S8). Taken together, these results indicate that the degradation of BRD7 by APC/C E3 ligase plays key roles in osteosarcoma.

### Inhibition of APC/C E3 complex suppresses cell proliferation through the APC/C-BRD7 pathway in osteosarcoma

**Fig 5:**
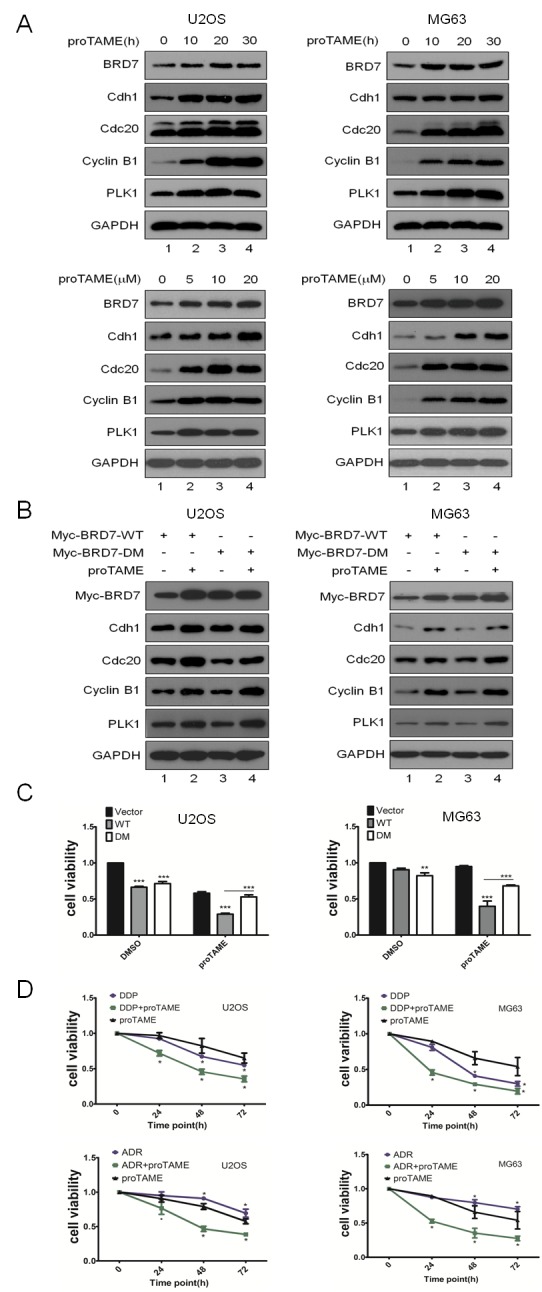
Inhibition of the APC/C E3 complex suppresses cell proliferation through the APC/C-BRD7 pathway in osteosarcoma The indicated cell lines were treated with different concentrations of proTAME at different time points as indicated, and the cells were then analyzed by Western blotting (n=3). (B) U2OS and MG63 cells were transiently transfected with BRD7-WT or BRD7-DM as indicated for 24 hrs and treated with 20 µM proTAME for another 20 hrs, and then the cells were analyzed by Western blotting (n=2). (C) Stable transfectants with BRD7-WT or BRD7-DM as indicated were treated with 20 µM proTAME for 48 hrs, and then the cell viability was measured by the MTT assay (n=3). * P<0.05 and ** P<0.001 using Student's t-test. (D) The indicated cell lines were treated with cisplatin (10 µM), doxorubicin (ADM, 40 ng/µl), or proTAME (15 µM) alone or a combination of two of the agents at different time points as indicated. Cell survival was measured using the MTT assay (n=3). * P<0.05 and ** P<0.001 using Student's t-test

Given that the APC/C-BRD7 pathway plays key roles in cell growth and the tumorigenesis of osteosarcoma (Fig. 4), which has a poor prognosis because of chemoresistance [15], we investigated whether apoptosis induced by chemotherapeutic drugs could be enhanced by inhibiting the APC/C-BRD7 pathway in osteosarcoma. Using cyclin B1, Cdh1, Cdc20 and Plk1 as positive controls, as shown in Fig. 5A, treatment with proTAME, a recently developed inhibitor of APC/C E3 ligase [22, 23], resulted in the stabilization of BRD7 in a time- and dose-dependent manner Furthermore, BRD7-WT, but not BRD7-DM, was stabilized by proTAME (Fig. 5B), and the stable transfectants with BRD7-WT were much more sensitive to cell proliferation inhibition by proTAME than those with BRD7-DM (Fig. 5C). More importantly, as shown in Fig. 5D, the combination of proTAME with commonly used chemotherapeutic drugs for osteosarcoma in the clinic, such as ADM and DDP, enhanced the killing effect in osteosarcoma cells. These results strongly indicate that inhibition of the APC/C-BRD7 pathway may increase the sensitivity of chemotherapeutic treatment for osteosarcoma.

### An inverse correlation was found between the protein levels of BRD7 and cdh1 or cdc20 in osteosarcoma tissues

**Fig 6:**
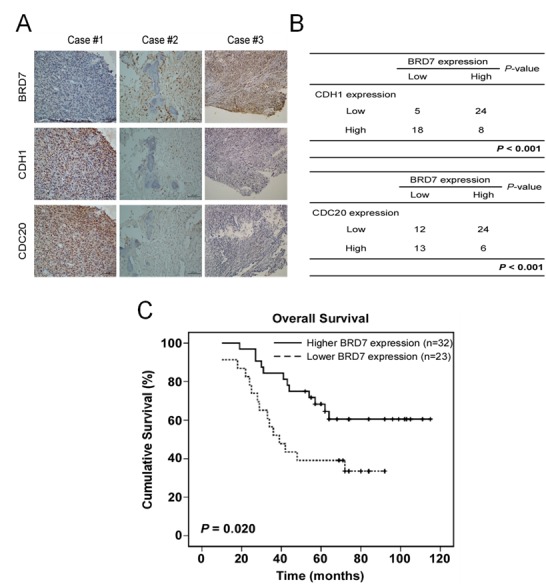
A lower expression of BRD7 indicates a poor prognosis, and there is an inverse correlation between the protein levels of BRD7 and CDH1 or CDC20 in human osteosarcoma (A,B) Immunohistochemical staining of BRD7, Cdh1 or Cdc20 was performed in the tumor tissues of 55 patients with osteosarcoma. Representative examples of BRD7 and Cdh1 or Cdc20 staining in the serial sections from the same tumor tissues are shown in (A), and a summary of the results is shown in (B). (C) Overall survival curves were generated based on the BRD7 protein expression statuses from these samples, and the actuarial probabilities were calculated using the Kaplan-Meier method and were compared using the log-rank test.

Finally, the significance of the interaction between BRD7 and Cdh1 or Cdc20 in human osteosarcoma tissues was determined by IHC using 55 samples whose characteristics were listed in Supplementary Table 1. Both Cdh1 and Cdc20 were mainly located in the nuclei of osteosarcoma cells (Fig.6A), consistent with the reports showing that the tumor cells exhibited positive nuclear staining of Cdh1 or Cdc20 [24-26]. Although BRD7 has been reported to be mainly localized in the nucleus [10], we found that BRD7 was mainly localized in the cytoplasm of osteosarcoma tissues. Furthermore, an inverse correlation was observed between the protein levels of BRD7 and Cdh1 or Cdc20 (p<0.001, χ^2^ tests, Fig.6B), demonstrating that Cdh1 and Cdc20 may also negatively regulate BRD7 protein in osteosarcoma tissues. Interestingly, there were some cases simultaneously containing high or low levels of Cdh1 and BRD7, or of Cdc20 and BRD7 (Fig. 6B). These results indicate that other pathways, in addition to the APC/C-BRD7 pathway, may be involved in the regulation of BRD7 protein levels in osteosarcoma.

Kaplan–Meier analysis and the log-rank test were used to evaluate the effects of BRD7 expression and clinicopathological characteristics on survival. Interestingly, BRD7 expression in osteosarcoma tissue was largely correlated with patients' survival time (p=0.020) (Fig. 6C). Lower or no BRD7 expression indicated a shorter overall survival time (OS) in patients with osteosarcoma compared with higher BRD7 expression.

## DISCUSSION

In the present report, we demonstrated for the first time that APC/C^cdh1^ and APC/C^cdc20^ regulate BRD7 protein stability and its anti-tumor function. In addition, we showed that the APC/C E3 ligase inhibitor proTAME sensitizes osteosarcoma cells to chemotherapeutic drugs. Importantly, the BRD7 protein levels are inversely correlated with Cdh1 or Cdc20 protein levels in osteosarcoma clinical samples. Furthermore, the lower level of BRD7 correlates with poor clinical outcome in patients with osteosarcoma.

BRD7, a member of the bromodomain protein family, has been shown to act as a tumor suppressor by binding the acetylated histones in chromosomes[27]. For example, down-regulation of BRD7 was reported in nasopharyngeal carcinoma and prostate cancer, and its inhibition of cell growth probably occurs through transcriptional regulation of ras/MEK/ERK, Rb/E2F, beta-catenin, which in turn affects progression of the cell cycle [10-12]. Our results showed that BRD7 may be regulated by APC/C E3 ligase during mitotic exit (Fig. 1A) and that the cell cycle profile was significantly changed by knockdown of BRD7 in osteosarcoma cells(Fig. S7). Interestingly, degradation of BRD7 by APC/C appears to be specific in osteosarcoma (Fig. 1D) because it was not observed in other types of cell lines tested (Fig. S3, S4). There are two possibilities, it is probably related to BRD7 localization in different cell types that depends on the post-translational modification of the protein[28]; BRD7 was mainly localized in the nuclei of nasopharyngeal carcinoma cells [10], whereas our IHC showed that BRD7 was mainly located in the cytoplasm of osteosarcoma tissue (Fig. 6A). In addition, SIRT2 has been recently reported to deacetylate Cdh1 and Cdc20, enhancing their binding with APC/C and indicating that acetylation may be involved in the regulation of the APC/C complex [29]. The acetylation status of Cdh1 or Cdc20 in osteosarcoma may be different from that in other cancer cell types, a topic that deserves future investigation.

Osteosarcoma is a rare type of tumor with poor prognosis in childhood and adolescence due to chemoresistance[15], making it necessary to identify new therapeutic targets for osteosarcoma. In this study, we found that APC/C^cdh1^ and APC/C^cdc20^ directly bind and degrade BRD7 (Figs. 1C, 2, and 3), a finding that is clinically relevant because a strong inverse correlation between the expression levels of BRD7 and Cdh1 or Cdc20 was observed in patients with osteosarcoma (Fig. 6B). Moreover, the BRD7 mutant that was resistant to APC/C degradation was more efficient at suppressing proliferation, colony formation and tumor growth in osteosarcoma (Fig. 4), and a lower level of BRD7 predicts poor prognosis in patients with osteosarcoma (Fig. 6C). These results demonstrated that BRD7 may function as a tumor suppressor in osteosarcoma, suggesting that targeting the APC/C-BRD7 pathway may be a promising strategy for treating osteosarcoma.

Notably, we found that proTAME, a recently developed inhibitor of APC/C E3 ligase[22, 23], can enhance the effect of chemotherapeutic drugs to kill osteosarcoma cells in vitro (Fig. 5D). Unfortunately, we could not further check the inhibitory effect of proTAME on tumor growth *in vivo*, because proTAME has not been tested for its usefulness in any animal model. Considering the xenograft nude mouse model with the same stable U2OS/MTX300 transfectants (Fig. 4D, 4E, 4F), we could expect that the combination of proTAME and chemotherapeutic drugs may be an attractive strategy for treating osteosarcoma.

**Fig 7:**
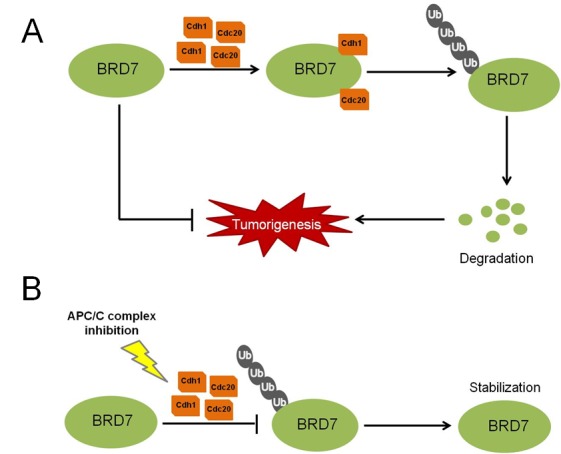
A proposed model for the regulation of BRD7 by APC/C E3 ligase in human osteosarcoma (A) High levels of Cdh1 or Cdc20 protein may promote the activity of APC/C E3 ligase and induce the degradation of BRD7, consequently favoring the tumorigenesis of osteosarcoma. (B) When APC/C is inhibited using small-molecule inhibitors, such as proTAME, the degradation of BRD7 is diminished, which in turn stabilizes BRD7 and consequently suppresses the tumor progression of osteosarcoma.

In summary, we propose a model for the regulation of BRD7 by the APC/C^cdh1^ and APC/C^cdc20^ E3 ligase complex in osteosarcoma (Fig. 7): two activators of the APC/C E3 ligase complex, Cdh1 and Cdc20, bind to BRD7 directly, triggering the degradation of BRD7 and consequently resulting in the loss of BRD7's inhibitory effects on the tumorigenesis of osteosarcoma. Conversely, when the APC/C complex is inhibited by small-molecule inhibitors, such as pro-TAME, the BRD7 protein is stabilized and suppresses osteosarcoma tumor progression.

## MATERIALS AND METHODS

### Cell lines

Three human osteosarcoma cell lines (U2OS, MG63, SAOS2) were cultured according to the instructions from the American Type Culture Collection (ATCC). U2OS/MTX300 cells were described previously [30]. All other cell lines (HeLa, HepG2, HEK293T, CNE-2, HCT116 and MCF-7) were cultured in Dulbecco's modified Eagle's medium (DMEM, Invitrogen) supplemented with 10% fetal bovine serum (HyClone), 1 mM glutamine and 100 U/ml each of penicillin and streptomycin.

### Plasmids

Full-length BRD7 cDNA was cloned into the pcDNA6/myc-His vector from human U2OS cells. GST-BRD7 was constructed by inserting BRD7 cDNA into the pGEX-4T-2 vector. Human Myc-Cdh1 and Myc-Cdc20 have been previously described [20, 31], and Flag- or HA-tagged Cdh1 and Cdc20 were generated by PCR amplification and sub-cloning into the pcDNA3.1 vector. GST-Cdh1 and GST-Cdc20 were constructed as above. Mutations were introduced using the Quick-Change Site-Directed Mutagenesis Kit (Stratagene), and all mutations were verified by DNA sequencing.

### Antibodies

The human anti-Cdh1 antibody was obtained from Abcam (Cat.# ab3242). Human anti-phospho-histone H3 (Ser 10) antibody was obtained from Millipore (Cat. #06-570). Other primary antibodies used for western blotting, which included anti-Cdc20 (SC-13162), anti-Cyclin B1 (SC-245), and anti-GAPDH antibodies (SC-166574), were obtained from Santa Cruz Biotechnology. Human anti-BRD7 antibody was purchased from Bethyl Laboratories, and anti-HA, anti-Flag, anti-Myc and anti-GST were from Cell Signaling Technology. Bound primary antibodies were detected with either horseradish peroxidase-conjugated anti-mouse IgG HRP or horseradish peroxidase-conjugated anti-rabbit HRP (Promega), and proteins were visualized by chemiluminescence.

For immunohistochemical staining, anti-BRD7 and anti-Cdc20 antibodies were purchased from Bethyl Laboratories, and the anti-Cdh1 antibody was obtained from Abnova.

### Transfection experiments

Transfection was performed as described previously [20, 32, 33]. Briefly, asynchronously growing cells seeded at 2.5 × 10^5^ cells per well in a 6-well plate or at 1 × 10^6^ cells per 10-cm plate were transfected with 2 µg or 12 µg of plasmid DNA, respectively, using LipofectamineTM 2000 (Invitrogen).

### RNA interference

The sequence of the BRD7 siRNA has been reported [13, 34]. The sequences of oligonucleotides1, 2 and 3, targeting BRD7 mRNA, were GUACUAAUGCCAUGAUUUA, GCACGUAUGGAGUUCGAAA and CGCUGAAAGCAGUAACAAATT, respectively. These siRNAs were synthesized by GenePharma. Knockdown of Cdc27 or APC2 was accomplished using SMARTpool siRNA(Dharmacon). Approximately 2 × 10^5^ U2OS cells per well were seeded in a 6-well tissue culture dish on the day before transfection. Transfection of 50 nmol siRNA was performed according to the manufacturer's instructions using the Lipofectamine™ RNAiMAX transfection reagent (Invitrogen). At 48 hrs after transfection, cells were incubated in the presence or absence of 25 µg/ml cycloheximide (CHX) for the indicated times and harvested in MCLB [50 mM Tris-HCl pH 8.0, 2 mM DTT, 5 mM EDTA, 0.5% Nonidet P-40, 100 mM NaCl, 1 mM microcystin, 1 mM sodium orthovanadate, 2 mM phenylmethanesulfonyl fluoride (PMSF), 1 ×protease (Sigma Chemical Co.) supplemented with a phosphatase inhibitor cocktail (Calbiochem)].

### RNA extraction and RT-PCR

These procedures were performed as previously described [30, 32, 35]. Briefly, total RNA was isolated using Trizol reagent (Invitrogen) according to the manufacturer's instructions. First-strand cDNA was synthesized using the Revert AidTM First Strand cDNA Synthesis Kit (MBI Fermentas). The primers used for amplifying BRD7, p21, MDM2 and GAPDH were as follows:

BRD7-F: 5'- TCTCTTGGGTCCCTCATACAG-3';

BRD7-R: 5'- CACTCAGCAACATCCGTCTT-3';

p21-F: 5'-GTGGGGTTATCTCTGTGTTAGGG-3';

p21-R: 5'-CCCTGTCCATAGCCTCTACTGC-3';

MDM2-F: 5'-GAATCTACAGGGACGCCATC-3';

MDM2-R: 5'-TCCTGATCCAACCAATCACC-3'

GAPDH-F: 5'-ACAGTCAGCCGCATCTTCTT-3';

GAPDH-R: 5'-GACAAGCTTCCCGTTCTCAG-3'.

### Synchronization of U2OS cells

This method was described previously [31-33]. Briefly, U2OS cells were cultured in the presence of 100 ng/mL nocodazole for 16hrs, and mitotic cells were isolated by mitotic shake off. To arrest cells at G1/S, cells were incubated in growth medium containing 2.5 mM thymidine for 17hrs. Cells were washed twice with PBS and placed in normal thymidine-free medium for 12hrs. Thymidine (2.5 mM) was added for a second 17hrs period, at which time cells were washed with PBS; this point was designated t=0. Next, cells were harvested and analyzed by flow cytometry, reverse transcription-polymerase chain reaction (RT-PCR), and western blotting, respectively.

### Flow cytometry

Flow cytometry was performed as described previously[32, 33]. Briefly, cells were harvested by trypsinization and collected by centrifugation. Cells were washed once with PBS and fixed in 1 ml of 70% ethanol at 4°C overnight. Cells were washed once with PBS/1% bovine serum albumin (BSA), and then incubated with 1 mL of PBS/1% BSA containing 30 µg/mL propidium iodide (PI) and 0.25 mg/mL RNase A for 30 mins at room temperature. Cells were analyzed for DNA content by flow cytometry using a Cytomics FC 500 flow cytometer (Beckman). The data were analyzed using Multicycle AV for Windows (Beckman).

### Western blotting and immunoprecipitation

Western blotting and immunoprecipitation were performed as described previously [32, 33]. Briefly, cells were lysed in MCLB, and clarified lysates were resolved by SDS-PAGE and transferred to PVDF membranes for western blotting using ECL detection reagents (Beyotime Co. Haimen, Jiangsu, China). Alternatively, clarified supernatants were first incubated with anti-Myc-agarose (Santa Cruz, SC-40AC), anti-FLAG-agarose (Sigma Chemical Co. A4596), or anti-HA-agarose (Sigma Chemical Co.) for 2 hrs to overnight at 4°C, and precipitates were washed four times with MCLB. To investigate the interaction between Cdh1 and BRD7, Cdc20 and BRD7 at the endogenous level, the clarified supernatants were first incubated with anti-Cdh1 or anti-Cdc20 for 2 hrs at 4 °C. Protein A/G-agarose was then added for 2 hrs to overnight, and precipitates were washed four times with MCLB and analyzed by Western blotting.

### GST pull down assays

GST, GST-BRD7, GST-Cdc20 and GST-Cdh1 were purified from bacteria using glutathione-agarose beads (GE Healthcare). Anti-Cdh1 or Cdc20 antibodies were used to immobilize GST-Cdh1 or GST-Cdc20 onto protein A beads and incubated with GST or GST-BRD7. After incubation overnight at 4°C, the beads were washed five times and bound proteins were analyzed by Western blotting.

### Colony formation assay and tumorigenicity assay

Colony formation assays were performed as previously described [30, 35, 36]. Briefly, U2OS cells were plated in triplicate at 100 cells per well in the 6-well plates. After culture with Dulbecco's modified Eagle's medium supplemented with 10% fetal bovine serum for 14 days, cell clones were washed three times with PBS, fixed in methanol for 10 mins, and dyed with crystal violet for 10 mins at room temperature. Thereafter, the dye was washed out, and colonies containing >50 cells were counted.

For tumorigenicity assays of the osteosarcoma cell lines, a total of 1×10^6^ U2OS/MTX300 cells stably transfected with empty vector, BRD7-WT and BRD7-DM (BRD7 double mutants), respectively, were suspended in 200 μl of PBS and injected subcutaneously into the mice, which were monitored for 8 weeks. The tumor size was measured using a sliding caliper every 3 days for 25 days using the formula V =1/2 (width^2^×length).

### Clinical samples

Patient studies were approved by the Institutional Review Board of Sun Yat-sen University, and written informed consent was obtained from the patients or their parents prior to sample collection. In the present study, 55 osteosarcoma samples were obtained. The ages of the patients ranged from 6 to 52 years. Tissue blocks prepared from osteosarcoma tissues and the adjacent tissues were sectioned for immunohistochemistry (IHC) to detect BRD7, Cdh1 and Cdc20 expression. The clinicopathologic features of the 55 patients are shown in Supplementary Table 1.

### Immunohistochemistry

This procedure was described previously [30, 35, 37]. Immunohistochemical analysis was performed on prepared3-μm sections. The primary antibodies against BRD7, Cdh1 and Cdc20 were diluted 1:250, 1:100 and 1:500, respectively, and were incubated at 4°C overnight in a humidified container. After washing with PBS three times, the tissue slides were treated with a non-biotin horseradish peroxidase detection system according to the manufacturer's instructions (Dako). IHC staining was evaluated by two independent pathologists who are experts in osteosarcoma pathology (Dr. An-Jia Han and Dr. Hui-Juan Shi). The protein expression levels of BRD7, Cdh1 and Cdc20 were classified as high level when the tumor tissue had more than 10% of cells positive for staining; otherwise, the expression levels were classified as low.

### Statistical analysis

Data were represented as the mean ± SD. Student's *t* test or the Mann-Whitney *U* test was employed to compare the values between subgroups. The association between Cdh1 or Cdc20 and BRD7 abundance was assessed using χ^2^ tests. Survival curves were constructed using the Kaplan-Meier method and compared using the log-rank test. Statistical analyses were performed using the SPSS 16.0 software (Chicago, IL). A *p* value less than 0.05 was considered significant; a *p* value less than 0.001 was considered strongly significant.

## SUPPLEMENTARY FIGURES AND TABLE


