# Supplementary material for: Targeting the anaphase-promoting complex/cyclosome (APC/C)- bromodomain containing 7 (BRD7) pathway for human osteosarcoma
Source: Oncotarget. 2014 Mar 21;5(10):3088–100. doi: 10.18632/oncotarget.1816 (PMC4102794; doi:10.18632/oncotarget.1816)
Supplement: Supplementary file 1 [file oncotarget-05-3088-s001.pdf]

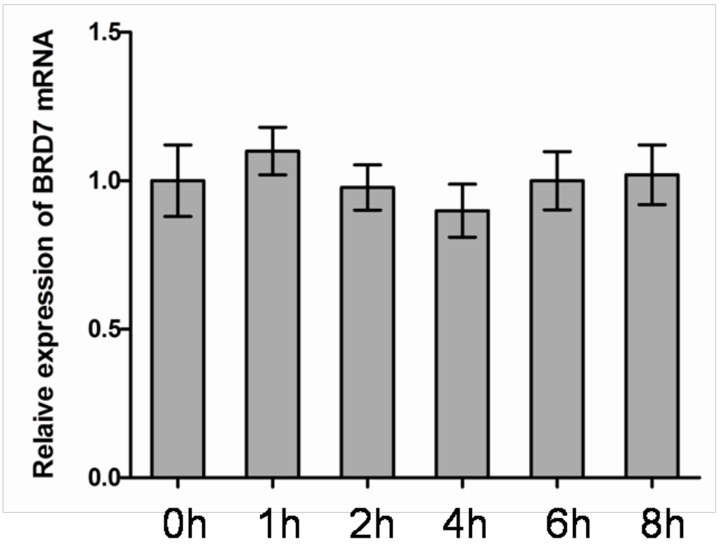


**Supplementary Figure S1. The mRNA level of BRD7 was constant in the cell cycle.** U2OS cells were arrested in mitosis by 18hrs of nocodazole treatment, released into fresh growth medium, and collected at the indicated times , the cells were subjected to RNA extracton for further RT-PCR analysis(n=3).

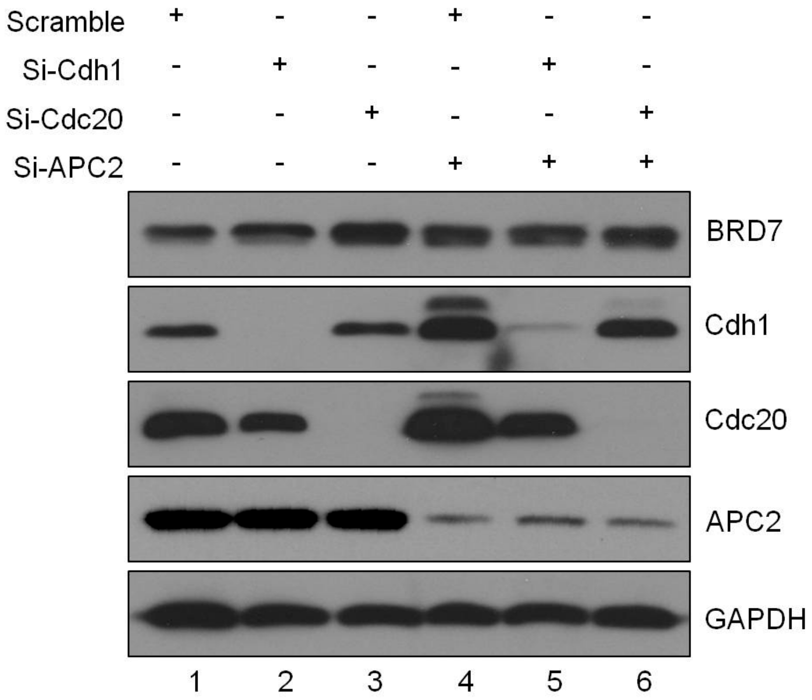


**Supplementary Figure S2. Knockdown of endogenous APC2 can also enhance BRD7 protein expression, and this increasing was not further enhanced by knockdown of endogenous Cdh1 or Cdc20.**

U2OS cells were transfected with siRNA, Cdh1 siRNA or Cdc20 siRNA alone, or together with APC2 siRNA. After incubation for 48 hrs, the cells were then analyzed as Fig.(1E) (n=3).

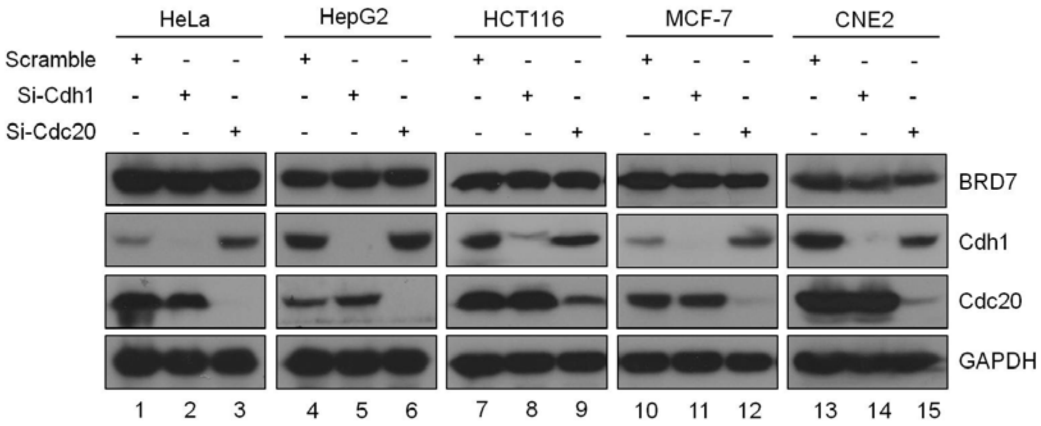


**Supplementary Figure S3. Knockdown of cdh1 or cdc20 does not induce the up-regulation of BRD7 protein level in other different types of tumor cells.** The cell lines of other different types of tumor HeLa, HepG2, HCT-116, MCF-7 and CNE-2 were transfected with scrambled , cdh1 or cdc20 siRNA as indicated for 48 hrs, and were analyzed as Fig.(1D)(n=3).

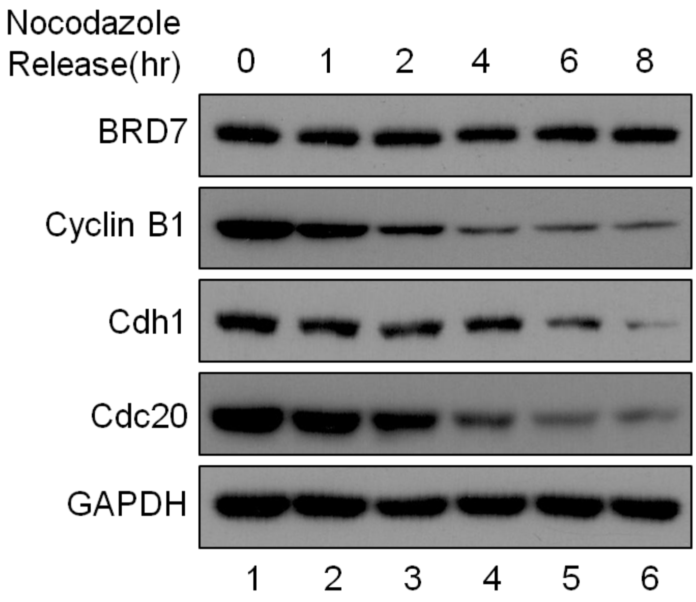


**Supplementary Figure S4. The protein Level of BRD7 was constant in the cell cycle of Hela cells.**

Hela cells were arrested in mitosis following 18 hrs of nocodazole treatment, released into fresh medium, and collected at the indicated times. The cells were harvested and subjected to Western blot analysis with the indicated antibodies (n=3).

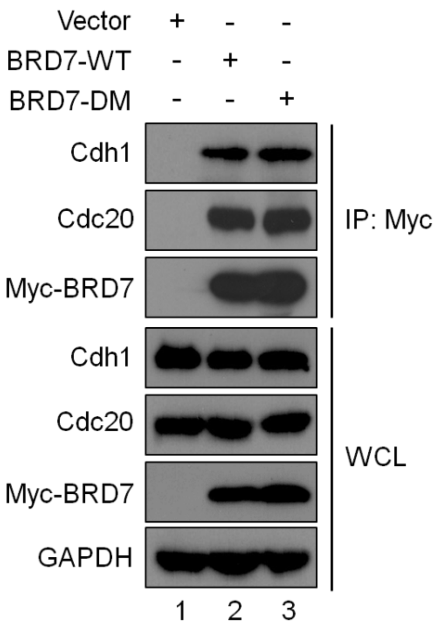


**Supplementary Figure S5. Both wild type BRD7(BRD7-WT) and mutant BRD7(BRD7-DM) have the same binding affinity with Cdh1 or Cdc20.** U2OS cells transfected with the indicated plasmids for 24 hrs were lysed with MCLB and subjected to IP using anti-Myc agarose and Western blotting with the indicated antibody were performed. WCL, whole cell lysate.

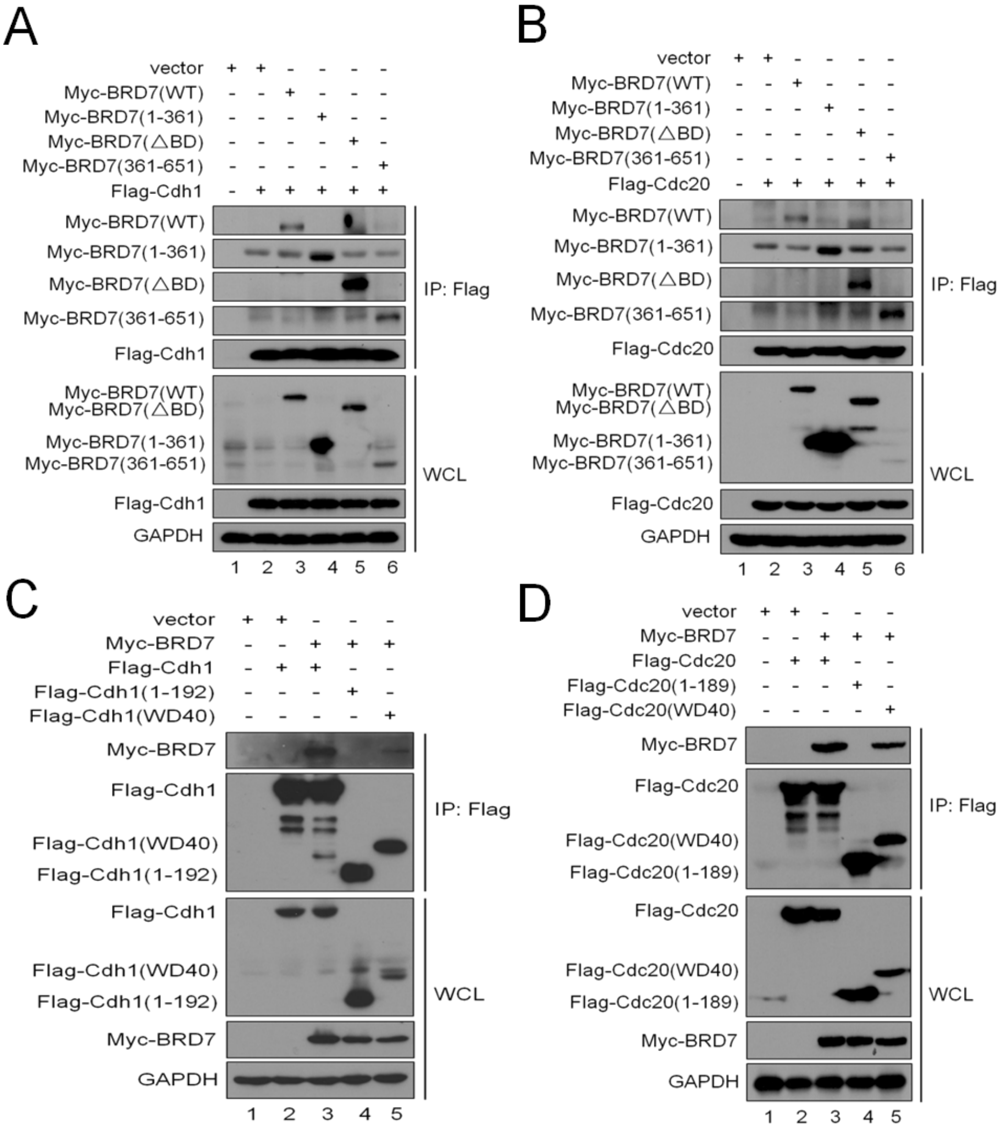


**Supplementary Figure S6. The interacting domain between BRD7 and Cdh1 or Cdc20.** (A,B) HEK293T cells co-transfected with Flag-Cdh1 or Flag-Cdc20 with various BRD7 mutants as indicated for 24 hrs were lysed with MCLB, and were analyzed as Fig.3A. (C,D) HEK293T cells co-transfected Myc-BRD7 with various Cdh1 or Cdc20 mutants as indicated for 24 hrs were lysed with MCLB, and were analyzed as Fig.3A.

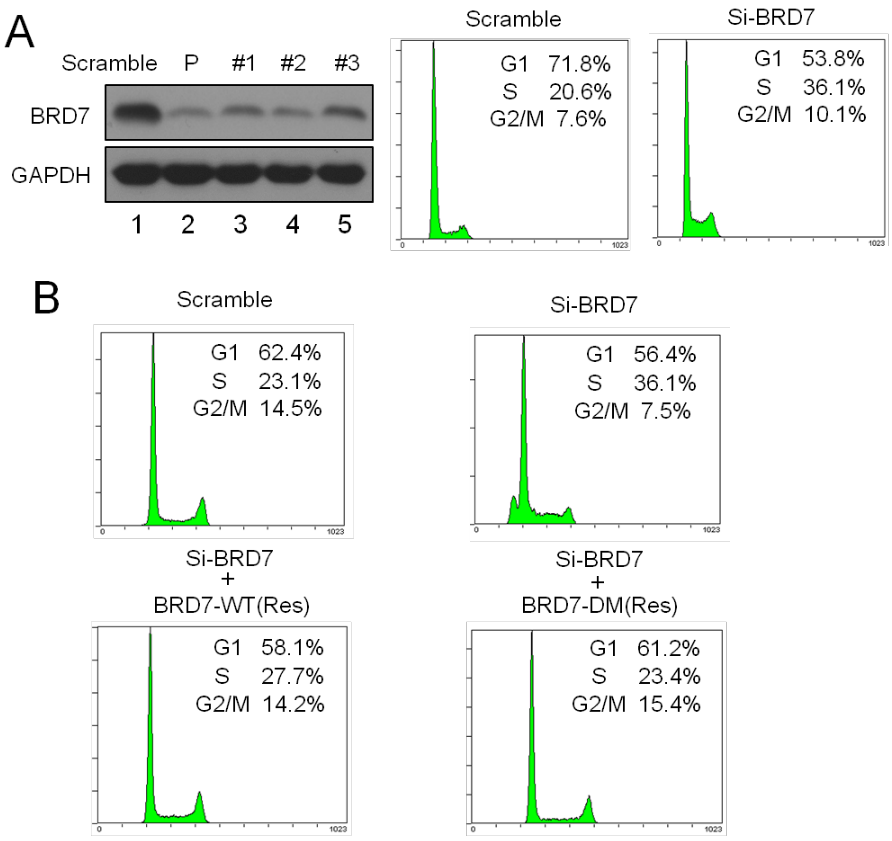


**Supplementary Figure S7. The mutant of BRD7 has higher effecificy than wildtype in inhibition of cell proliferation.** (A) U2OS cells were transfected with scrambled or BRD7 siRNAs as indicated for 48 hrs and collected the cells, one part of the cells were used for protein extraction and subjected to Western blotting(left pannel) ; the rest of the cells were stained with propidium iodide and analyzed by folw cytometry (right pannel), P : a pool mixed with equal amount of three individual BRD7 siRNAs) . (B) U2OS cells were transfected with scramble or siRNA for 24 hrs, then re-transfected with BRD7-WT(Res) or BRD7-DM(Res) plasmid for 24 hrs, then harvested the cells and suffered to FACS analysis.

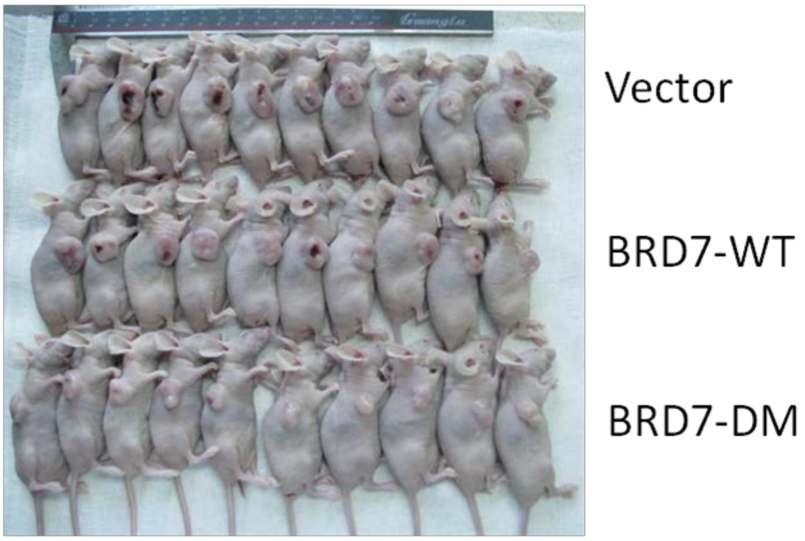


**Supplementary Figure S8. The xenograft nude mouse model with indicated treatment.** Tumorigenicity of the indicated stable transfectants when grown for 5 weeks in the nude mouse model (n = 10 mice per group).

**Supplementary Table 1**

Patient characteristic at initial diagnosis.

| Characteristics | No. of patients (%) |
|---|---|
| Gender | |
| Male | 34(61.8%) |
| Female | 21(38.2%) |
| Age(years) | |
| Median(range) | 17(6-52) |
| Anatomic site | |
| Femur | 35(63.6%) |
| Tibia | 13(23.6%) |
| Humerus | 2(3.6%) |
| Fibulars | 2(3.6%) |
| Ulna | 1(1.8%) |
| Ilium | 2(3.6%) |
| Histologic type | |
| Osteoblastic | 25(45.5%) |
| Chondroblastic | 4(7.3%) |
| Fibroblastic | 8(14.5%) |
| Telangiectatic | 1(1.8%) |
| Not specified | 17(30.9%) |
| Enneking staging | |
| ⅡB | 51(92.7%) |
| Ⅲ | 4(7.3%) |
